# ADAMTS Gene-Derived circRNA Molecules in Non-Small-Cell Lung Cancer: Expression Profiling, Clinical Correlations and Survival Analysis

**DOI:** 10.3390/ijms25031897

**Published:** 2024-02-05

**Authors:** Jacek Pietrzak, Rafał Świechowski, Agnieszka Wosiak, Szymon Wcisło, Ewa Balcerczak

**Affiliations:** 1 Laboratory of Molecular Diagnostics and Pharmacogenomics, Department of Pharmaceutical Biochemistry and Molecular Diagnostics, Medical University of Lodz, Muszynskiego 1, 90-151 Lodz, Poland; jacek.pietrzak@umed.lodz.pl (J.P.); rafal.swiechowski@umed.lodz.pl (R.Ś.); agnieszka.wosiak@umed.lodz.pl (A.W.); 2 BRaIn Laboratories, Medical University of Lodz, Czechoslowacka 4, 92-216 Lodz, Poland; 3 Department of Thoracic, General and Oncological Surgery, Medical University of Lodz and Military Medical Academy Memorial Teaching Hospital of the Medical University of Lodz - Central Veteran Hospital, 90-542 Lodz, Poland; szymon.wcislo@umed.lodz.pl

**Keywords:** ADAMTS, circRNA, non-small-cell lung carcinoma, regulation of transcription

## Abstract

The present study examines the relationship between circular RNA (circRNA) derived from three genes of the family a disintegrin and metalloproteinase with thrombospondin motifs (ADAMTSs): *ADAMTS6*, *ADAMTS9* and *ADAMTS12* and the host gene expression in non-small-cell lung cancer (NSCLC) with regard to various clinical factors. Notably, an association was identified between *ADAMTS12* expression and specific circRNA molecules, as well as certain expression patterns of *ADAMTS6* and its derived circRNA that were specific to histopathological subtypes. The survival analysis demonstrated that a lower *ADAMTS6* expression in squamous cell carcinoma was associated with extended survival. Furthermore, the higher *ADAMTS9* expression was linked to prolonged survival, while the overexpression of *ADAMTS12* was correlated with a shorter survival. These findings suggest that circRNA molecules may serve as potential diagnostic or prognostic biomarkers for NSCLC, highlighting the importance of considering molecular patterns in distinct cancer subtypes.

## 1. Introduction

Among cancer types, the leading cause of death is non-small-cell lung cancer (NSCLC). Despite advances in treatment, the prognosis of patients is usually poor, and a 5-year survival is less than 15% [1]. In almost 80% of NSCLC cases, oncogenic driver mutations were identified, which primarily included the *KRAS*, *EGFR*, *BRAF*, *MET*, *ERBB2*, *ALK*, *RET*, *ROS1* and the *NTRK1/2/3* genes [2]. Other genetic factors regulating the expression of genes responsible for proliferation, apoptosis or the immune response in the development of lung cancer are also disturbed.

One of the important genetic regulatory factors are ncRNA molecules, such as microRNA (miRNA), long non-coding RNA (lncRNA) and circular RNA (circRNA) [3]. CircRNA molecules do not usually directly encode protein structures, but perform numerous regulatory functions. They are formed by joining the 5′ and 3′ ends of gene transcript fragments together to form a circular structure [4]. CircRNAs can act as miRNA-binding sponges, preventing them from attaching to complementary mRNA, thus affecting the expression of many genes, including host genes. CircRNAs also bind numerous transcription and regulatory proteins, affecting their availability. In addition, circRNA can be translated, thus increasing protein concentration [5]. The level of circRNA expression will therefore significantly regulate proliferation, apoptosis, differentiation and aging, as well as the innate immune response and maintenance of stem cell pluripotency [6]. Although CircRNA was until recently regarded as an abnormal product of the pre-mRNA maturation process, devoid of biological function, it has since been confirmed as a regulatory factor of many cellular processes, and a possible route for transmitting signals to neighboring cells.

It is important to note that, in the course of cancer, changes occur in both the cell from which the cancer originates and the micro-environment surrounding the cancer tissue (TME) [7]. These changes can be used as potential markers indicating the clinical stage and subtype of the cancer, but also for predicting the response to treatment and its outcome. Tumor development is characterized by extracellular matrix (ECM) remodeling, in which a crucial role is played by proteolytic enzymes, whose activity is dependent on the presence of metal ions in their active site. These enzymes include metalloproteinases (MMPs), disintegrin and metalloproteinase (ADAM), a disintegrin and metalloproteinase with thrombospondin motifs (ADAMTSs) [8]. ADAMTS proteins play a crucial role in cancer by influencing the various aspects of the tumor microenvironment. These proteins are involved in the degradation of extracellular matrix (ECM) components, impacting tissue architecture and promoting tumor cell invasion. Certain ADAMTS proteins, such as ADAMTS1 and ADAMTS12, have been implicated in angiogenesis, the formation of new blood vessels that support tumor growth. Additionally, ADAMTS proteins can modulate the activity of growth factors and cytokines, contributing to tumor cell proliferation and survival. The ADAMTS expression or activity is often dysregulated in various cancer types, highlighting their potential as therapeutic targets for cancer treatment [9].

The main goal of the study was to evaluate the expression of five circRNA molecules formed on the DNA template of the *ADAMTS6* (*hsa_circ_0004418*, *hsa_circ_0072676*), *ADAMTS9* (*hsa_circ_0066444*) and *ADAMTS12* (*hsa_circ_0006624*, *hsa_circ_0072119*) genes in lung tissue collected from patients with NSCLC. The second aim of the project was to evaluate the expression of the *ADAMTS6*, *ADAMTS9* and *ADAMTS12* genes in the tumor tissue samples. The selection of circRNA molecules was based on data gathered from the “CircFunBase” database, which indicated their involvement in cancer [10]. The tested circRNA molecules based on data collected in the CircFunBase database were previously only analyzed in gastric cancer tissue. The results of this analysis indicated the overexpression of the tested circRNAs in gastric cancer tissue compared to normal tissue [11,12]. However, only in the case of *hsa_circ_0066444* was it associated with an unfavorable prognosis for patients [12]. Therefore, the conducted research is one of the few in which selected circRNA molecules were analyzed.

## 2. Results

### 2.1. Correlation between the Expression of circRNA Molecules and Their Host Genes

**Figure 1:**
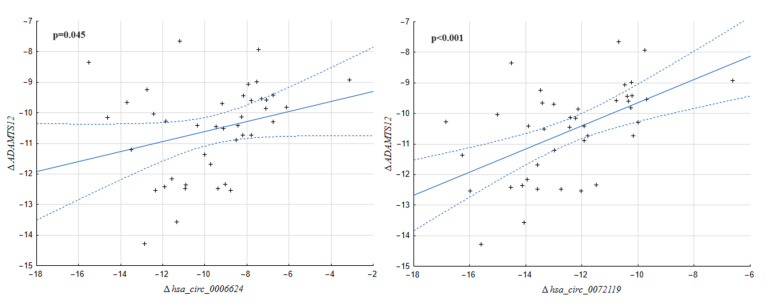
Correlation between the expression of the ADAMTS12 gene and those of *hsa_circ_0006624* (*p* = 0.045) and *hsa_circ_0072119* (*p* < 0.001). The regression band marked as a dashed line indicates a 0.95 confidence interval.

The assessment of the correlation between the expression level of the *ADAMTS6*, *ADAMTS9* and *ADAMTS12* genes and the expression level of circRNA molecules based on these genes turned out to be interesting. Although no relationships were found for *ADAMTS6* and *hsa_circ_0004418* (*p* = 0.589) and also *hsa_circ_0072676* (*p* = 0.338) or *ADAMTS9* and *hsa_circ_0066444* (*p* = 0.056), correlations were confirmed only between *ADAMTS12* and *hsa_circ_0006624* (*p* = 0.045), as well as *hsa_circ_0072119* (*p* < 0.001) (Figure 1).

### 2.2. Expression of circRNAs and Their Host Genes in Relation to the Histopathological Subtype of NSCLC

**Figure 2:**
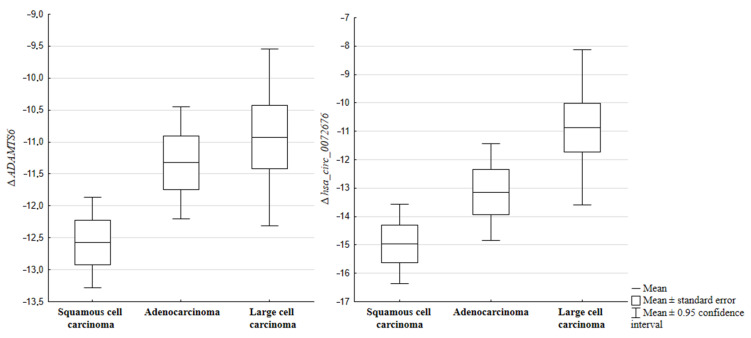
The relationship between the expression of the *hsa_circ_0072676* (*p* = 0.0243) gene, ADAMTS6 (*p* = 0.0329) and its host gene with regard to the histopathological subtype of NSCLC.

Then, the correlation between the histopathological subtype and the expression level of the tested genes and circRNA molecules was evaluated. Statistical analysis revealed the differences in the expression level of the *ADAMTS6* gene. Squamous cell carcinoma was characterized by a lower mRNA level of the *ADAMTS6* gene than adenocarcinoma (*p* = 0.0329). Another difference was the expression level of *hsa_circ_0072676*, which was also statistically significantly higher in large-cell lung cancer compared to squamous cell carcinoma (*p* = 0.0243) (Figure 2).

### 2.3. Expression of circRNAs and Their Host Genes in Relation to Tumor Progression according to the TNM Classification

The presence of a relationship between the size of the primary tumor [T] according to the TNM classification and the expression level of all tested genes and all circRNA molecules was checked. There was no correlation between the expression of all examined circRNA molecules and its parental genes according to the primary tumor size. The next analyzed element was the assessment of the interaction between the presence of metastases to regional lymph nodes [N] and the expression level of the *ADAMTS6*, *ADAMTS9* and *ADAMTS12* genes. Similarly to the previous analysis, there was no correlation between the expression levels of all tested genes, circRNA molecules and the involvement of regional lymph nodes by cancer cells. Next, the impact of the presence of distant metastases on the expression level of the studied genes and circRNA molecules was analyzed. In this case, there was also no correlation between the presence of distant metastases and the expression level of the tested genes and circRNA molecules.

### 2.4. Expression of circRNAs and Their Host Genes in Relation to the Infiltration of Surrounding Tissues

**Figure 3:**
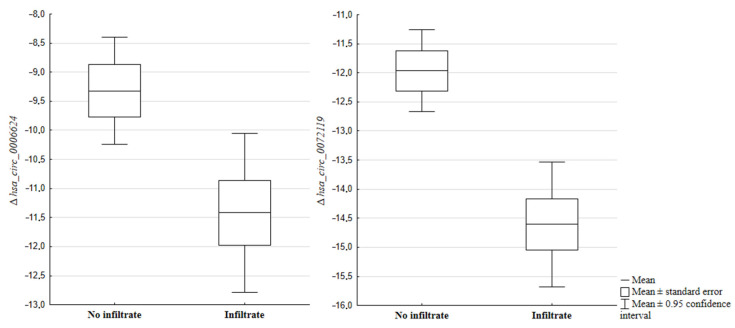
Differences in *hsa_circ_0006624* (*p* = 0.0389) and *hsa_circ_0072119* (*p* = 0.0021) gene expression levels depending on the presence of infiltration of surrounding tissues.

Interesting results were obtained from the comparative analysis of expression levels of the tested genes and circRNA molecules and the presence/absence of infiltration the surrounding tissues. A lower expression level of *hsa_circ_0006624* (*p* = 0.0389), *hsa_circ_0072119* (*p* = 0.0021) was found in tumors that infiltrated the surrounding tissues (Figure 3). For the remaining circRNA molecules, *hsa_circ_0004418* (*p* = 0.0519), *hsa_circ_0072676* (*p* = 0.433), *hsa_circ_0066444* (*p* = 0.5539) as well as their host genes *ADAMTS6* (*p* = 0.7795), *ADAMTS9* (*p* = 0.4530) and *ADAMTS12* (*p* = 0.7730), no similar relationship was observed.

### 2.5. Comparison of the Expression Levels of the Genes ADAMTS6, ADAMTS9 and ADAMTS12 between Tumor Tissue from NSCLC Patients with Histological Subtype Division and Normal Lung Tissue

**Figure 4:**
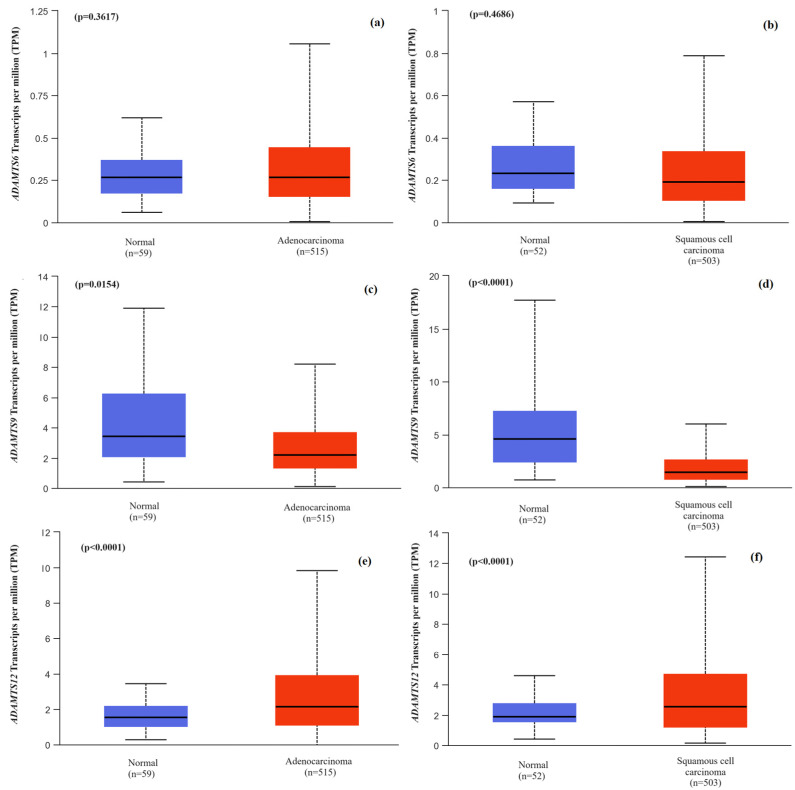
Box plots comparing the expression of genes *ADAMTS6*, *ADAMTS9* and *ADAMTS12* in tumor tissue from NSCLC patients and normal tissue: (**a**) *ADAMTS6* expression in adenocarcinoma compared to normal tissue; (**b**) *ADAMTS6* expression in squamous cell carcinoma compared to normal tissue; (**c**) *ADAMTS9* expression in adenocarcinoma compared to normal tissue; (**d**) *ADAMTS9* expression in squamous cell carcinoma compared to normal tissue; (**e**) *ADAMTS12* expression in adenocarcinoma compared to normal tissue; (**f**) *ADAMTS12* expression in squamous cell carcinoma compared to normal tissue. The analysis was performed using data collected in the external UALCAN database.

Based on data collected from the UALCAN database, differences in the *ADAMTS6*, *ADAMTS9* and *ADAMTS12* gene expression levels were also assessed between tumor tissue and normal tissue. The analysis revealed no statistically significant differences in the *ADAMTS6* expression between normal tissue and adenocarcinoma (*p* = 0.3617) or squamous cell carcinoma (*p* = 0.4686). Conversely, distinct results were obtained for the expression of the *ADAMTS9* gene. It was observed that both adenocarcinoma (*p* = 0.0154) and squamous cell carcinoma (*p* < 0.0001) exhibited a lower expression of the *ADAMTS9* gene compared to normal lung tissue. The expression of the *ADAMTS12* gene was also compared between normal and tumor tissue, revealing differences between these two materials. However, in contrast to the expression of the *ADAMTS9* gene, the expression of the *ADAMTS12* gene was overexpressed in tumor tissue, both in adenocarcinoma (*p* < 0.0001) and squamous cell carcinoma (*p* < 0.0001). The results are presented in Figure 4.

### 2.6. Survival Analysis Based on the Expression of ADAMTS6, ADAMTS9 and ADAMTS12 in the Tumor Tissue of Patients with NSCLC

**Figure 5:**
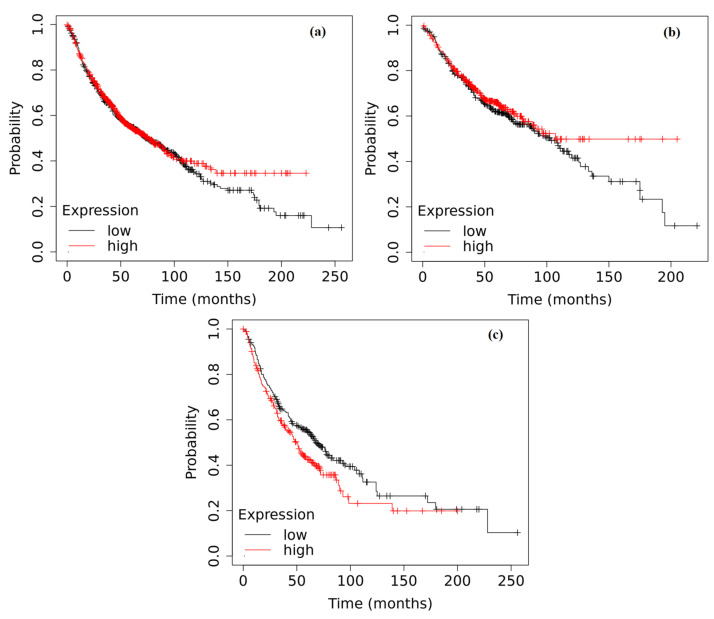
Kaplan–Meier curves depicting the survival analysis of NSCLC patients based on the expression of the *ADAMTS6* gene in tumor tissue: (**a**) Patients without histopathological subtype division (*p* = 0.4622); (**b**) Adenocarcinoma (*p* = 0.2618); (**c**) Squamous cell carcinoma (*p* = 0.0224). The analysis was performed based on data collected in the Kaplan–Meier plotter database.

A survival analysis was conducted based on the *ADAMTS6*, *ADAMTS9* and *ADAMTS12* expression in tumor tissue using data gathered from the external Kaplan–Meier Plot database. The analysis included all NSCLC samples without histopathological subclassification and considered the two most common histological subtypes of adenocarcinoma and squamous cell carcinoma. The analysis revealed highly interesting results. The *ADAMTS6* expression did not significantly affect the overall survival of NSCLC patients without the histopathological subtype division (*p* = 0.4622) or the adenocarcinoma patients (*p* = 0.2618). However, among squamous cell carcinoma patients, a lower expression of *ADAMTS6* was associated with longer survival compared to those with higher expression (*p* = 0.0224) (Figure 5).

**Figure 6:**
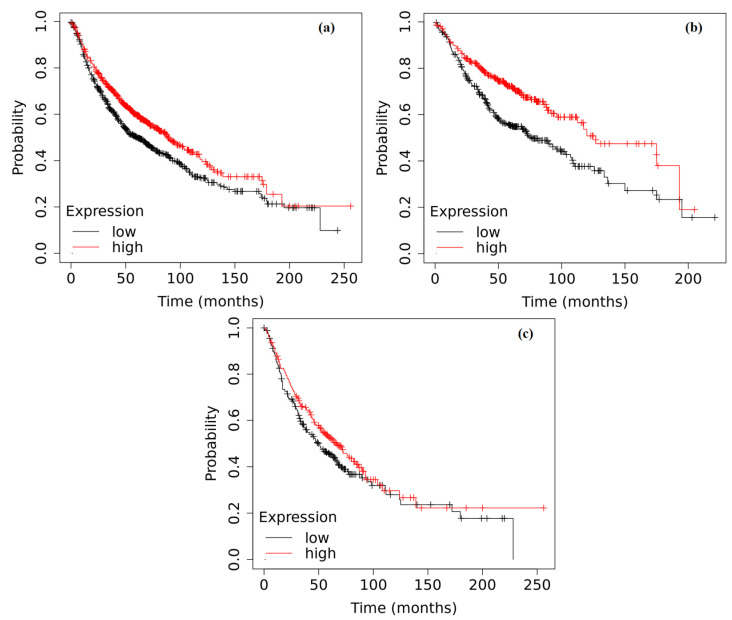
Kaplan–Meier curves depicting the survival analysis of NSCLC patients based on the expression of the *ADAMTS9* gene in tumor tissue: (**a**) patients without histopathological subtype division (*p* = 0.0006); (**b**) adenocarcinoma (*p* < 0.0001); and (**c**) squamous cell carcinoma (*p* = 0.1029). The analysis was performed based on data collected in the Kaplan–Meier plotter database.

Completely different results were obtained regarding the influence of *ADAMTS9* gene expression on survival in NSCLC patients. Briefly, a significantly higher level of *ADAMTS9* gene expression correlated with a longer survival in NSCLC patients without a division into histopathological subtypes (*p* = 0.0006). This correlation was particularly pronounced in the adenocarcinoma subtype (*p* < 0.0001), where the survival of patients with a higher *ADAMTS9* gene expression (127 months) was almost twice as long as that of individuals with lower expression (76 months). Interestingly, the *ADAMTS9* gene expression appeared to have no significant impact on survival duration in patients with squamous cell carcinoma (*p* = 0.1029) (Figure 6).

**Figure 7:**
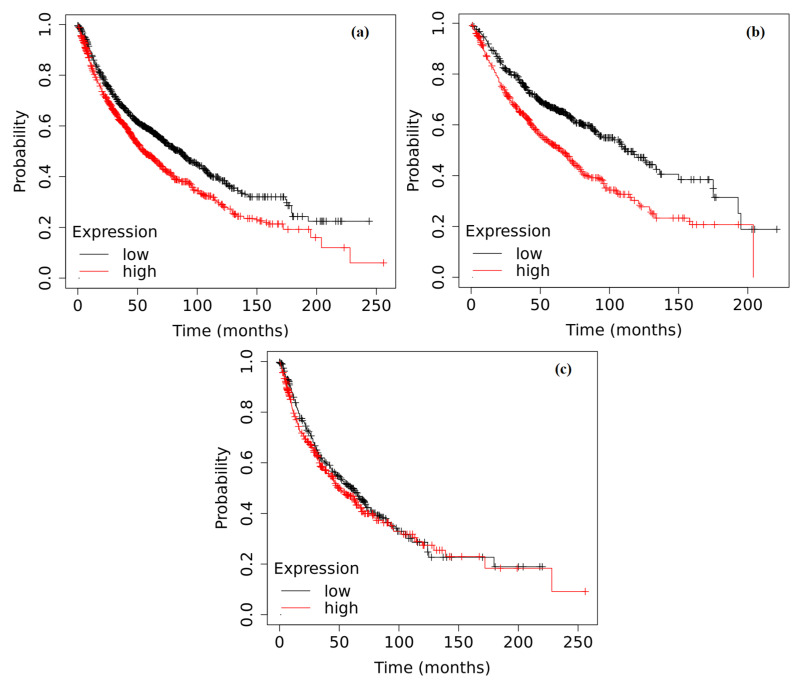
Kaplan–Meier curves depicting the survival analysis of NSCLC patients based on the expression of the *ADAMTS12* gene in tumor tissue: (**a**) patients without histopathological subtype division (*p* < 0.0001); (**b**) adenocarcinoma (*p* < 0.0001); (**c**) squamous cell carcinoma (*p* = 0.3183). The analysis was performed based on data collected in the Kaplan–Meier plotter database.

The analysis of the impact of *ADAMTS12* gene expression levels on survival proved to be equally intriguing. *ADAMTS12* gene expression correlated with a shorter survival in NSCLC patients (*p* < 0.0001); similarly, the higher expression of the *ADAMTS12* gene in patients with adenocarcinoma was associated with an almost two-fold reduction in survival time, i.e., from 113 months for a lower expression to 65 months for a higher expression (*p* < 0.0001). However, similar to *ADAMTS9*, the *ADAMTS12* gene expression did not appear to have any significant effect on survival duration in squamous cell carcinoma (*p* = 0.3183) (Figure 7).

### 2.7. Analysis of Interactions between the Tested circRNA Molecules and miRNAs and mRNA of Genes Encoding ADAMTS Family Proteins, including Host Genes

The analysis of possible interactions between the examined circRNA molecules and mRNA coding for ADAMTS family protein genes, including host genes, was conducted based on data gathered from external databases. The analysis was grounded in the properties of circRNA as miRNA sponges, which resulted in the reduction in miRNA levels by binding to these molecules. Consequently, this leads to a lower availability of miRNA molecules capable of binding to mRNA, causing a limitation in mRNA degradation. This results in increased transcript stability and may be associated with the overexpression of various genes, ultimately leading to elevated protein concentration.

**Figure 8:**
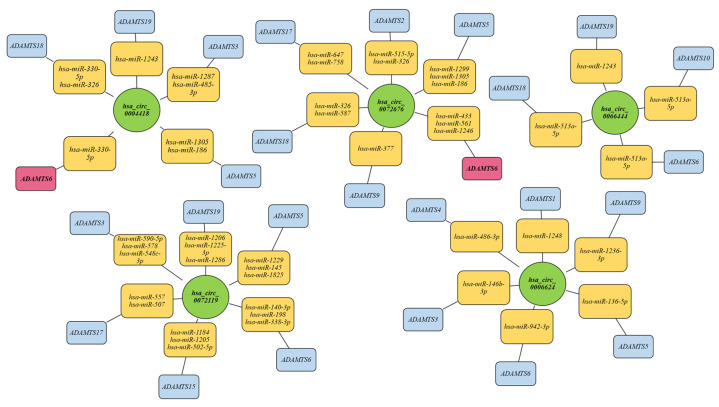
circRNA/miRNA/mRNA interaction network predicted using the CircInteractome database miRDB and miRTarBase. Host genes that are regulated by circRNAs created on their matrix are marked in red rectangles.

The analysis began with an assessment of the potential interactions between the examined circRNA molecules and potential miRNA molecules. It was demonstrated that all circRNA molecules can bind to numerous miRNA molecules, including those that interact with the mRNA of ADAMTS family proteins. However, only the circRNA molecules originating from the *ADAMTS6* gene matrix showed the ability to bind to miRNA molecules that directly regulate the expression of this gene. The results of the analysis are presented in Figure 8.

## 3. Discussion

The mammalian genome produces a huge number of non-coding transcripts [13]; it is estimated that over 90% of the human transcriptome is not translated [14]. However, these molecules are not devoid of biological function; they mainly control the process of gene expression [15]. Most importantly for the clinician, the expression of non-coding RNAs is tissue-specific, and may be a potential marker of disease, including cancer.

Numerous studies have confirmed that circRNA performs important biological functions in the functioning of cells, including those that have undergone cancer transformation. CircRNA molecules are transcription regulators that control the expression of genes involved in proliferation, apoptosis [7] and angiogenesis [16], all of which play important roles in oncogenesis. CircRNAs can regulate the availability of various mRNAs by functioning as sponges that bind miRNA molecules [17]. Additionally, some circRNA molecules can act as templates for the translation process, with the resulting proteins potentially having biological activity [18,19]. Moreover, circRNA molecules have been found to be very stable and conserved structures: mouse and rat genomes have been found to share 15,000 circRNA molecules with humans [18]. Hence, circRNA may become important diagnostic or prognostic markers for cancer in the future.

The presented work examined the expression of five circRNA molecules created on the DNA template of the *ADAMTS6* (*hsa_circ_0004418*, *hsa_circ_0072676*), *ADAMTS9* (*hsa_circ_0066444*) and *ADAMTS12* (*hsa_circ_0006624*, *hsa_circ_0072119*) genes from the lung tissue of NSCLC patients; it also assessed the expression of its host genes. It determined the effect of various clinical and demographic conditions on the expression of the tested gene structures. Only one correlation was found, i.e., between *ADAMTS12* expression and both tested circRNA and their host genes: no similar relationship was found for *ADAMTS6* or *ADAMTS9*. The bioinformatic analysis, however, indicated that only in the case of circRNA molecules whose host gene was *ADAMTS6*, was a potential interaction of circRNA/miRNA/mRNA found which could influence the stability of the *ADAMTS6* transcript. However, this finding was not reflected in the course of the studies, where no correlation was observed between *hsa_circ_0004418*, *hsa_circ_0072676* and the expression of *ADAMTS6*. In the case of *hsa_circ_0066444*, *hsa_circ_0006624* and *hsa_circ_0072119*, similar potential interactions could not be confirmed. Some circRNA molecules regulate the expression of their parental genes, one of the important mechanisms by which circRNA molecules influence cell functioning. Some circRNA molecules regulate the expression of their parental genes, which is one of the important mechanisms by which circRNA molecules influence cell functioning. For example, Li et al. found that *circITGA7* or its parent gene *ITGA7* (integrin subunit alpha 7) knockdown enhanced proliferative and metastatic potential in colorectal cancer [20]. Li et al. also confirmed the presence of *circ_MMP2* in the exosomes secreted by hepatocellular cells into normal liver cells; the presence of *circ_MMP2* enhanced the tumorigenesis and metastasis via sponging miR-136-5p, which subsequently led to an increased expression of the *MMP2* (matrix metalloproteinases 2) gene [21]. Richardson et al. found circRNA molecules to play a role in regulating the expression of the *PTEN* (phosphatase and tensin homolog) gene which is important in carcinogenesis processes. The *circPTEN* molecule acts as a molecular sponge for miR-155 and miR-330-3p, which inhibit cell growth and inactivates the carcinogenic PI3K/AKT signaling pathway in non-small-cell lung cancer by increasing expression of the *PTEN* gene [22].

Our findings indicate that the expression of the *ADAMTS6* gene and the derived circRNA molecule, *hsa_circ_0072676*, varies considerably between the histological subtypes of non-small-cell lung cancer (NSCLC). Moreover, the survival time of patients, which, in some cases, correlated with the expression levels of the studied genes, also depended on the histological subtype of the tumor. Non-small-cell lung cancer is a heterogeneous disease, and its prognosis and choice of treatment are largely connected with the histopathological subtype and the presence of changes in the cancer cell genome [23]. Furthermore, environmental factors have varying impacts on the occurrence of specific tumor subtypes. For instance, tobacco smoking is strongly associated with squamous cell carcinoma. Additionally, specific subtypes are characterized by different mutations: adenocarcinoma typically exhibits changes in the *EGFR* and *ALK* genes, while squamous cell carcinoma is associated with alterations in the *PIK3CA* and *FGFR1* genes. Large-cell carcinoma, often classified in cases not assigned to adenocarcinoma or squamous cell carcinoma, shows mutations characteristic of NSCLC, such as *KRAS* and *TP53* [24]. The molecular basis of NSCLC subtypes is diverse, leading to distinct alterations in genes responsible for apoptosis, proliferation and extracellular matrix remodeling. Therefore, individual modifications in gene expression regulation should be interpreted based on the NSCLC subtype.

Bioinformatic analysis revealed a decrease in the expression of the *ADAMTS9* gene and an increase in *ADAMTS12* expression in the cancer tissue of patients with NSCLC compared to normal tissue. Survival analysis further demonstrated that this molecular pattern, i.e., a decrease in *ADAMTS9* expression and an increase in *ADAMTS12* expression, is associated with shorter survival. Interestingly, these results align with findings from the changes in circRNA expression in the tumors invading surrounding tissues, whereas a lower expression of *hsa_circ_0006624* and *hsa_circ_0072119* was associated with the presence of invasion into surrounding tissues. ADAMTS proteins exhibit properties that both favor and inhibit the cancer process [25]. In cell models, Koo et al. demonstrated anti-angiogenic properties for ADAMTS9 [26] and El Hour et al. have demonstrated anti-angiogenic properties in cell models for ADAMTS12 [27]; however, the underlying mechanisms for these effects seem to involve different processes. Du et al. also found that the overexpression of *ADAMTS12* is linked to reduced Akt and mTOR phosphorylation in gastric cancer and that the knockdown of *ADAMTS-9* increases the tumorigenic potential in colorectal and breast cancer [28]. On the other hand, Fontanil et al. demonstrated that overexpression of *ADAMTS12* enhances the metastatic potential in breast cancer [29]. Additionally, circRNA molecules appear to negatively interact with the expression of host genes in breast cancer: the increased expression of *circSMARCA5* lowered the expression of the *SMARCA5* gene, consequently inhibiting DNA repair [30].

## 4. Materials and Methods

The materials consisted of 61 tissue fragments of non-small-cell lung cancer (NSCLC) collected intraoperatively. The research was conducted with the approval of the Bioethics Committee (No. RNN/87/16/EC and KE/952/22). The clinicopathological characteristics and smoking status of the study group are presented in Table 1.

### 4.1. RNA Isolation

Total RNA was isolated from tissues using the miRNeasy Micro Kit (Qiagen, Hilden, Germany) according to the attached protocol. After isolation, the concentration and purity of the isolated RNA was checked spectrophotometrically. Samples with A260/A280>1.9 and concentration >250 ng/µL were qualified for further analyses.

### 4.2. Reverse Transcription

Reverse transcription was performed with 1 µg of isolated RNA using the High-Capacity cDNA Reverse Transcription Kit High-Capacity cDNA Reverse Transcription Kit (Applied Biosystems™, Waltham, MA, USA) according to the attached protocol.

### 4.3. qPCR

Quantitative real-time PCR was conducted with *ADAMTS6* (Hs01552731_m1), *ADAMTS9* (Hs00172025_m1), *ADAMTS12* (Hs00917098_m1) specific molecular probes (Applied Biosystems™, USA) and TaqMan Fast Advanced Master Mix (Applied Biosystems™, USA) on a QuantStudio™ 5 Real-Time PCR System (Applied Biosystems™, USA). *GAPDH* (Hs02758991_g1) and *ACTB* (Hs01060665_g1) were used as reference genes. All experiments were carried out in duplicate. The relative expressions of *ADAMTS6*, *ADAMTS9* and *ADAMTS12* were calculated as the difference between the obtained Ct value for the tested genes and the arithmetic mean of the reference genes.

### 4.4. Degradation of Linear RNA

The linear RNA molecules were degraded using RNase R (ab286929) (Abcam, Cambridge, Great Britain, UK). Briefly, 20U RNase R, 40U RiboLock RNase Inhibitor (Thermo Scientific™, Waltham, MA, USA) and reaction buffer were added to 10 µg of the previously isolated total RNA. Following this, the reaction mix volume was made up to 20 µL with RNase free water, and the mixture was incubated for 150 min at 37 °C. After incubation, the remaining RNA molecules were purified using the miRNeasy Micro Kit (Qiagen, Germany) according to the attached protocol. After purification, reverse transcription was performed using the Thermo Scientific Maxima First Strand cDNA Synthesis Kit for RT-qPCR (Thermo Scientific™, USA) according to the attached protocol.

### 4.5. qPCR for circRNA

Power SYBR™ Green PCR Master Mix (Applied Biosystems™, USA) was used to quantify the expression of the tested circRNA molecules in accordance with the attached protocol. All experiments were carried out in triplicate. The primer sequences used to identify the individual circRNA molecules were designed in NCBI primer-BLAST [31] (Table 2).

### 4.6. Analysis of the External Database

Differences in the expressions of the *ADAMTS6*, *ADAMTS9* and *ADAMTS12* genes between tumor and normal tissue were assessed using data collected in the external UALCAN database [32,33]. Samples examined in the TCGA program (The Cancer Genome Atlas Program) were selected for comparison. Survival was assessed with regard to *ADAMTS6*, *ADAMTS9* and *ADAMTS12* gene expression in tumor tissue based on data collected in the Kaplan–Meier plotter database [34]. Tumor cases were analyzed as adenocarcinoma or squamous cell carcinoma, and as NSCLC as a whole. To assess the impact of the studied circRNA molecules on the expression levels of host genes, as well as other genes in the ADAMTS protein family, data from the CircInteractome database [35] were utilized. This database allowed the prediction of miRNA molecules that could bind to the studied circRNA, rendering them inaccessible to mRNA molecules. For evaluating the interactions between miRNA and mRNA molecules, miRDB [36,37] and miRTarBase [38] databases were employed. This approach facilitated the creation of a network depicting the relationships between the studied circRNA molecules and the expression of genes in the ADAMTS protein family, including host genes.

### 4.7. Statistical Analysis

The statistical analysis was performed using Statistica 13.1 software (TIBCO, Palo Alto, CA, USA). The Shapiro–Wilk test was used to confirm the normality of continuous distributions. Student’s *t*-test and one-way ANOVA test were used to analyze differences between individual subpopulations. The log-rank test was used to analyze survival rates in general populations. Pearson correlation was used to assess the correlation between quantitative variables. In all the subsequent calculations, the significance was set at the level of 0.05.

## 5. Conclusions

Circular RNA molecules have a significant influence on the functioning of both normal and cancer cells. The present study examined changes in the expression of five circRNA molecules whose parental genes belong to the ADAMTS protein family, which is involved in remodeling the extracellular matrix. The results confirm that changes in the circRNA quantity are associated with the expression of their parental genes and clinical characteristics. Future research should include potential protein structures that may arise from circRNA matrices. These studies should be accompanied by an assessment of the miRNA molecules capable of binding to the examined circRNA, as they can also significantly influence cell functioning. Nevertheless, given its functions and molecular stability, circRNA may become a potential diagnostic or prognostic biomarker for cancer, including non-small-cell lung cancer.

## Figures and Tables

**Table 1:** Clinical characteristics of the study group.

Clinical Parameter	Number of Patients			
Smoking tobacco products	Yes—40		No—21	
Pack–year	Mean—39.69 (SD—18.33)			
Histological subtype	Sqamous cell carcinoma 31	Adenocarcinoma 20	Large-cell carcinoma 5	Other 6
Primary tumor size (T in TNM classification)	T1—12	T2—23	T3—16	T4—10
Lymph node involvement (N in TNM classification)	N0—16	N1—27	N2—16	N3—2
Presence of distant metastasis (M in TNM classification)	Mx—25	M0—30		M1—6
Another cancer	Yes—12		No—59	

**Table 2:** Primer sequences used for assessing the expression of the investigated circRNA.

Primer Name	Sequence
*hsa-ADAMTS6 0002* forward	5′-CTGTGACAGTCCAGCGTAAGT-3′
*hsa-ADAMTS6 0002* reverse	5′-TGACACAGCGGTTGCTTTTG-3′
*hsa-ADAMTS6 0003* forward	5′-GTGACAGTCCAGCACCTTCAG-3′
*hsa-ADAMTS6 0003* reverse	5′-ACGCTTGGGAGGCTCATTAT-3′
*hsa-ADAMTS9 0002* forward	5′-GACGCTGCATGGAGTACTGG-3′
*hsa-ADAMTS9 0002* reverse	5′-GACATACAACAACACGCCGC-3′
*hsa-ADAMTS12 0001* forward	5′-CTCAGTGGCACGGTTCTACA-3′
*hsa-ADAMTS12 0001* reverse	5′-TCTACTCGGACTGGACCCAC-3′
*hsa-ADAMTS12 0004* forward	5′-CCTCCTTTCCTGCAACAGAGA-3′
*hsa-ADAMTS12 0004* reverse	5′-GCGGCCCTTCTTTATGCAATG-3′

## Data Availability

The data presented in this study are available on request from the corresponding author.
